# Assessing needs of patients and families during the perioperative period at King Abdullah Medical City

**DOI:** 10.1186/s13741-020-00141-9

**Published:** 2020-04-07

**Authors:** Wid Alsabban, Ahmed Alhadithi, Faisal Salem Alhumaidi, Abdullah Mutlaq Al Khudhair, Saeed Altheeb, Ahmed Saad Badri

**Affiliations:** 1 Perioperative Medicine Administration, King Abdullah Medical City-Makkah, Makkah, Saudi Arabia; 2 College of Medicine, King Khalid University, Abha, Saudi Arabia; 3 College of Medicine, Umm Al Qura University, Makkah, Saudi Arabia; 4 College of Medicine, Imam Abdularhman bin Faisal University, Dammam, Saudi Arabia

**Keywords:** Patient satisfaction, Patient experience, Perioperative, Communication, Family needs

## Abstract

**Background:**

This paper explores elective surgery patients’ and family members’ needs during the perioperative period, in a specialized hospital in Saudi Arabia. Needs are influenced by context and could differ from a setting to another.

**Methods:**

Two questionnaires, one for the patient group and the other for the family member group, were adopted from a previous similar study. The participants were asked to rate the importance of each need and how much it was satisfied. Data were collected in 5 weeks. Descriptive statistics were used to determine the average rate and standard deviation of each item.

**Results:**

Patients highly rated the need for adequate symptom management in the recovery area. Family members highly rated the importance of being informed if the surgical procedure is taking more time than expected and communicating with the surgeon after the procedure.

**Conclusion:**

Systematically involving the family member in the perioperative care of the patient is advantageous. However, interventions and extent of involvement of the family member to the care of the patient would have to be adapted according to the cultural context.

## Background

The perioperative time is a stressful period for both the patient and the family member (Leske 1993; Mark 2003). Understanding their prioritized needs is essential to improve their experience. Because prioritized needs and satisfaction factors differ culturally, it is important to explore them in local settings (Halligan 2006).

In the Kingdom of Saudi Arabia (KSA)—similar to other middle eastern cultures—families form strong ties, and they are an essential source for social, emotional and spiritual support of the patient (Saleh Al Mutair et al. 2014). For example, family members would address health professionals for information about the patient and could influence decision making (Halligan 2006; Almutairi et al. 2015). Also, a family member is expected to stay with the patient in the hospital, and family members would be receiving and facilitating breaking the bad news to the patient (Saleh Al Mutair et al. 2014). Concordantly, it was found that patients are inclined toward family involvement in their healthcare rather than an autonomous patient approach when their perceptions were explored in a local hospital in KSA (Mobeireek et al. 2008). Nonetheless, the boundaries of the role of family members as part of the care providers in the hospital are confusing to healthcare professionals, particularly to nurses (Halligan 2006; Alshahrani et al. 2018).

The conflicting cultural values and ambiguity of family member boundaries to expatriate nurses was an impetus to explore cultural competence in healthcare in Saudi Arabia (Almutairi et al. 2015). KSA is a vast country with a 32,000,000 population, and the majority are youth (WHO n.d.). To meet the healthcare needs of this growing population, a large number of expatriate nurses joined the healthcare workforce (Aldossary et al. 2008). Consequently, research initiatives to explore and utilize the concept of cultural competence were undertaken and emphasized the unique cultural challenge of nursing-family member relationship (Almutairi et al. 2015; Alshahrani et al. 2018; Almutairi and Rondney 2013). For example, nurses were worried about the constant presence of the family members as sometimes they pose a risk on patient safety as the family member is trying to take care of the patient’s needs (Alshahrani et al. 2018). Nurses also felt emotionally stressed about family members involved in decision making that sometimes put the patients through a lot of unnecessary interventions (Halligan 2006). The studies on cultural competence raised the importance of a family-centred approach in healthcare in Saudi Arabia (Almutairi and Rondney 2013).

In the perioperative setting, the role of family members had been recognized on a global level (Shields 2007). Actual integration started initially at paediatric care and extended to the adult perioperative setting (Smykowski and Rodriguez 2003). The high measured level of anxiety of both the patient and the family member in the perioperative period was a significant factor in developing patient and family-centred interventions to address stressors (Mark 2003; Dexter and Epstein 2001). One example is the postanaesthesia care unit (PACU) visits of family members, the visits were found to improve satisfaction and anxiety for both patients and family members (Carter et al. 2012). Furthermore, anxiety levels of family members could improve with in-person progress report while waiting for the patient in surgery, especially when the surgical time is extended (Stefan 2010; Blum and Burns 2013).

Given the cultural context that reinforced family involvement and the stress on both family and patient in the perioperative setting, it is crucial to explore their needs in the Saudi environment. We did not find any studies done in Saudi Arabia that examined the needs of patients and family members during the perioperative period. We also did not find any example for a model of family-centred care in healthcare in KSA in any healthcare setting. For this purpose, in this study, we aimed to survey and describe the prioritized perceived needs of patients and their family members who were admitted for elective surgery in KAMC and asses their level of satisfaction. We will contrast that to established needs in western societies and how it might differ to needs in KSA.

## Methods

### Study design

A descriptive cross-sectional survey study design was used to assess the prioritized perioperative needs of the patients and accompanying family members after elective surgery.

The study was conducted in the perioperative administration at King Abdullah Medical City (KAMC), Makkah, Saudi Arabia. KAMC is a tertiary and quaternary governmental healthcare centre. We gained ethical approval from the Institutional Review Board at KAMC before starting the data collection process.

Our targeted population was all patients who were admitted for elective surgery and were sent back to the ward after surgery and their accompanying family members. A convenience sampling was used. Participants were selected based on a list of patients who have been received in the recovery area. Our population was the whole number of patients who met the criteria during the time of the study. The reason for that is that the flow and type of patient would differ throughout the year, especially with pilgrimage seasons. Population number was estimated to be 215 participants in total. With a confidence interval of 95% and a margin of error of 10%, the sample size would be 67 participants from each group. This was accepted in alignment with the exploratory nature of the study (Denscombe 2014).

All patients and family members were approached to survey the day after surgery in their rooms at KAMC from 24 of July to 24 of August 2017. The investigators introduced themselves as they were not staff members of KAMC and took the consents for participation in the study. The investigators explored with the participants the benefits from their participation in the study with emphasis on confidentiality as the survey did not include any nominative information. The investigators then asked them to fill the questionnaire and added any further needs that were not included in the survey. The questionnaire took not more than 5 min for completion. The investigators were present with the participants to support them fill the questionnaire and were present when the questionnaire is being answered so that they would attend to any needs for clarifications.

### Inclusion/exclusion criteria

Our inclusion criteria for patients and accompanying family members were as follows: to be 18 years or more of age, the patient was admitted for elective surgery and the ability to cooperate and communicate with the investigator. We excluded participants who were unable to speak Arabic, same-day surgery patients and patients who were sent to ICU after surgery.

### Data collection tool

Two similar questionnaires were used one for the patient participant and the other for the accompanying family member participant. These questionnaires were adapted from a previous study and translated (Davis et al. 2014). The reason we adapted these questionnaires is that they were the only that we found that would assess the needs of the participant for the whole perioperative period. The questionnaires were reviewed by two nurses, were also linguistically reviewed and were piloted on four others who are none healthcare professionals. Each questionnaire had two groups of questions concerning the needs of the participants. In the first question, the participant was asked to rate the need by a four-level Likert scale regarding importance. In the second question, the participant was asked to determine how much the need was met. Also, questions about logistical information before coming to the hospital and demographic data were collected. We were limited to the items of the survey, though some spaces for comments were left. The survey would allow for baseline knowledge on how such needs might differ in our local community and support further research and exploration.

The patient’s questionnaire had 26 needs, and the family member’s questionnaire had eighteen needs. These needs were grouped into time periods in the perioperative time. There was a space to comment after each time period in the questionnaire. The questionnaire’s items focused on the following: communication with hospital staff, information giving and physical comfortability. There have been some patients and family members who refused to participate in our study (estimated No. 5–10 participants). The reasons why they did not want to be part of the study were as follows: they were not educated enough to understand the content, were too sick to participate in the study, or simply did not have time to fill the survey.

Data was inserted electronically into SPSS. Importance of each need was assigned a numerical code for coding purposes: 0 = not important at all, 1 = not important, 2 = important and 3 = very important. Moreover, whether the item was met or not was assigned a numerical value for coding purposes: 0 = not met, 1 = partially met and 2 = met. Descriptive statistics were used to reflect patients and family member needs and experience. The average means and standard deviation were calculated for each need, and the needs were ranked accordingly. Missing data was amputated from the calculation.

## Results

We have surveyed 144 participants over 5 weeks; 77 of them were patients, while 67 were family members. The total response rate among participants in the study was around 85 %. The mean age of patients was 49.58 (SD = ± 16.3) years, while the mean age of family members was 35.03 (SD = ± 9.83) years. The vast majority of participants were Saudis, with diverse educational levels. Family member participants were 38.8% of the time the sons or daughters of the patients. About 80–90% of patients and family members reported that they were informed about the time of admission. 51.9% of patients and 67.2% family members reported receiving information before coming to the hospital about the location for admission, and 55.8% of patients received information on taking their usual medications before admission. The majority of patients and accompanying family members reported that they had not received any information before coming to the hospital about where to park, items to bring to the hospital and the location of the family waiting area (see Table 1).

**Table 1:** Characteristics of patient and family member participants

**Characteristics**	**Patients (*N*= 77)**	**Family members (*N*= 67)**
**Age (years) mean ± (SD)**	49.58 ± (16.3)	35.03 ± (9.83)
**Gender**		
Female	50	43
Male	27	24
**Nationality**		
Saudi	65	58
Non-Saudi	12	9
**Highest educational level completed**		
Non educated	18	4
Elementary school	11	7
Junior high school	11	3
High school	14	25
Diploma	7	2
Bachelor	13	21
Advanced study	3	4
**Received information before coming to the hospital on**		
	**Yes**	**Yes**
Time to arrive	89.6%	80.6%
Where to park	19.5%	34.3%
Location for admission	51.9%	67.2%
Items to bring to hospital	24.7%	43.3%
Information on taking your usual medications before coming to the hospital	55.8%	ــــــــــــــ
Location of OR family room	22.1%	53.7%
**Relationship to patient**		
Father or mother	10	
Husband or wife	4	
Brother or sister	15	
Son or daughter	26	
Others	10	
Missing	2	

Patients’ mean scores for responses to the importance of each need and how well these needs were met during each time period within the perioperative period are summarized in Table 2. The overall top-ranked needs for patients in the perioperative period are as follows: being treated with respect by hospital personnel, adequate symptom management by the recovery room staff and physical comfortability of the recovery area. Mean scores for how well patient needs were met during the perioperative experience were 1.5 out of 2 for all surveyed needs

**Table 2:** Patients average (±SD) scores for importance of perioperative needs and how often those needs were perceived to have been met

**Patient member need (item number refers to order in original survey)**	**Importance of need**	**Rank order of need importance in each time period**	**Was need met?**
**Time period: Before coming to the hospital**			
Having information on how your pain will be managed during and after the surgery.	2.57 ± (0.59 )	1	0.91 (0.92)
Having information about the surgical procedure itself (e.g. how the surgery is done, complications, expected hospitalization time).	2.55 ± (0.80 )	2	1.44 (0.78)
Having someone answer your questions before you come to the hospital.	2.11 ± (0.78)	4	0.90 (0.86)
Having information about what to do on the day of surgery (e.g. time to arrive, where to park, where to go in the hospital, what to bring).	2.40 ± (0.74 )	3	0.80 (0.92)
**Time period: Day of surgery in the preoperative surgical care area**			
Being treated with respect by hospital personnel (e.g. personnel protected your privacy and modesty, addressed you in a courteous manner, took time to understand and answer your questions).	3.00 ± (0.00)	1	1.95 (0.40)
Having information about the procedures for getting you ready for surgery (e.g. admission assessment, starting an IV infusion, getting medications before surgery, movement by cart to the operating room).	2.37 ± (0.74)	7	1.26 (0.79)
Having your family member or significant other with you in the presurgical area.	2.53 ± ( 0 .90)	6	1.51 (0.68)
Being physically comfortable (bed, room temperature, noise and activity level in the room, management of your pain or other symptoms)	2.78 ± (0.42)	2	1.71 (0.59)
Having important information about you communicated to hospital personnel before your admission.	2.64 ± (0.60)	4	1.70 (0.68 )
Having opportunities to ask questions and address concerns with hospital staff.	2.61 ± (0.47)	5	1.82 (0.55)
Having hospital staff reassure you about any fears/anxieties you might have related to your surgical experience.	2.78 ± (0.36)	2	1.68 (0.74)
Being informed about delays in the operating room schedule.	2.77 ± (0.63)	3	1.35 (0.91 )
**Time period: Day of surgery in the operative room area**			
Having important information about you communicated to hospital personnel before your arrival in the operating room.	2.25 ± (1.14)	6	1.38 ± (0 .92 )
Being treated with respect by hospital personnel (e.g. personnel protected your privacy and modesty, addressed you in a courteous manner, took time to understand and answer your questions).	2.93 ± (0.17)	1	1.97 ± (0.24)
Having hospital staff reassure you about any fears/anxieties you might have related to your surgical experience.	2.61 ± (0.76)	3	1.76 ± (0.50)
Having opportunities to ask questions and address concerns with hospital staff.	2.39 ± (0.74)	4	1.68 ± (0.77)
Being physically comfortable (on bed, room temperature, noise and activity level around you).	2.71 ± (0.74)	2	1.79 ± (0.57)
Having information about the role of operating room staff (nurse, technician, anesthesiologist).	2.35 ± (0.86)	5	1.45 ± (0.80)
**Time period: Post-anaesthesia care unit area**			
Having your pain, nausea, and/or vomiting adequately managed by the recovery room staff.	2.83 ± (0.37)	2	1.56 ± (0.68)
Having information about your condition.	2.72 ± (0.54)	4	1.56 ± (0.72)
Being treated with respect by hospital personnel (e.g. personnel protected your privacy and modesty, addressed you in a courteous manner, took time to understand and answer your questions).	2.91 ± (0.53)	1	1.91 ± (0.54)
Having important information about you communicated to hospital personnel before your arrival in the recovery room.	2.19 ± (0.89)	7	1.36 ± (0.90)
Being physically comfortable (bed, room temperature, noise and activity level in the room).	2.78 ± (0.58)	3	1.83 ± (1.68)
Having opportunities to ask questions and address concerns with hospital staff.	2.18 ± (1.06)	8	1.48 ± (0.87)
Reassurance from hospital staff about fears/ anxieties related to the surgical experience.	2.54 ± (0.91)	5	1.50 ± (0.88)
Having your family member or significant other visit you in recovery room.	2.22 ± (1.12)	6	1.07 ± (0.93)

Family members’ mean scores for responses to the importance of each need item and how well these needs were met during each time period within the perioperative period are summarized in Table 3. The overall top-ranked needs for family members in the perioperative period are as follows: being treated with respect by hospital personnel, having communication with the surgeon or other physicians after the procedure and being informed about delays in the operating room schedule. Mean scores for how well family members’ needs were met during the perioperative experience were 1.4 out of 2 for all surveyed needs.

**Table 3:** Family members’ average (±SD) scores for importance of perioperative needs and how often those needs were perceived to have been met

**Family member need (item number refers to order in original survey)**	**Importance of need**	**Rank order of need importance in each time period**	**Was need met?**
**Time period: Before coming to the hospital**			
Having information about the surgical procedure itself (e.g. how the surgery is done, complications, expected hospitalization time).	2.9 (0.42 )	1	1.46 (0.87)
Having information about what to do on the day of surgery (e.g. time to arrive, where to park, where to go in the hospital, what to bring).	2.58 (0.63 )	3	1.11 (0.85)
Having someone answer your questions before you come to the hospital.	2.59 (0.60 )	2	1.03 (0.97)
**Time period: Day of surgery in the preoperative area and during the operation**			
Having communication with the surgeon after the procedure with consideration for privacy.	2.91 (0.29)	3	1.54 (0.86 )
Having opportunities to ask questions and address concerns with hospital staff.	2.82 (0.41)	6	1.63 (0.64 )
Being with your family member/significant other while they were in the preoperative surgical care area.	2.85 (0.44)	4	1.71 (0.70)
Being treated with respect by hospital personnel (e.g. personnel protected your privacy, addressed you in a courteous manner, took time to understand and answer your questions).	2.97 (0.24)	1	1.80 (0.46)
Having communication with operating room nurses about the patient’s condition during the surgical procedure.	2.80 (0.61)	7	1.47 (0.79 )
Being informed about delays in the operating room schedule.	2.92 (0.33)	2	1.47 (0.82)
Having information about the presurgical care procedures for getting the patient ready for surgery (e.g. admission assessment, starting an intravenous infusion, getting medications before surgery, movement by cart to the operating room).	2.77 (0.46)	5	1.53 (0.71)
Being physically comfortable in the OR family room area.	2.68 (0.69)	8	1.07 (0.98)
**Time period: Day of surgery in the post anaesthesia care unit area**			
Having communication with the surgeon or other physicians after the procedure with consideration for privacy.	2.92 (0.33)	2	1.46 (0.76)
Having opportunities to ask questions and address concerns with hospital staff.	2.88 (0.39)	3	1.51 (0.76)
Having communication with recovery room nurses about the patient’s condition during the surgical procedure.	2.76 (0.61)	5	1.28 (0.94)
Being treated with respect by hospital personnel (e.g. personnel protected your privacy, addressed you in a courteous manner, took time to understand and answer your questions).	2.93 (0.29)	1	1.84 (0.52)
Visiting or being with your family member/significant other while they were in the recovery room.	2.48 (0.97)	7	1.09 (0.98)
Being informed about when transfer to a regular hospital room would take place.	2.81 (0.36)	4	1.58 (0.74)
Being physically comfortable in the OR family room area.	2.70 (0.69)	6	1.21 (0.87)

Very few participants wrote in the comment sections. Repeated comments were concerning the unavailability of a waiting area for the family members during the surgical operation time.

## Discussion

In this study, we explored and described the prioritized needs of patients and their family members who were admitted for elective surgery in KAMC. For this purpose, a questionnaire that was used in a previous similar study was adopted and translated to fit the intended context (Davis et al. 2014).

The needs listed in the questionnaire were all averagely rated to be at least important. The needs were partially satisfied by the patients and their family members on average, indicating a room for improvement and further exploration of those needs and their attributes. The lowest-ranked need was given an average rate of 2.11 of importance in a 4-point Likert scale (very important [3], important [2], not important [1], not important at all [0]). Our result shows that the top three most important needs from the perspective of the patient are as follows: adequate symptom management in the recovery area along with physical comfortability of its environment, and reassurance by a healthcare provider just before surgery and communication if the surgery schedule was delayed. On the other hand, our results demonstrate that the highly ranked needs from the family members’ perspective are as follows: being informed about the surgical procedure, communication if the surgery is taking longer than expected and reassurance from the surgeon once the procedure is done. The top need for both patients and family members, namely “being treated with respect by hospital personnel” were not considered here in the discussion due to acquiescence bias. This is explained by the nature of the question asking them about the rating importance of respectful treatment. All participants graded this need as highly important in the survey.

We found that the literature described interventions to tackle patients’ and family members’ anxiety that are relevant to the top-ranked needs in our results. In the following, we would list these interventions and their correlated needs:

First, preoperative education programme with information on postoperative symptom management and information about the surgical procedure itself could improve the anxiety level of patients and improve symptom management postoperatively (Garretson 2004; Kiyohara et al. 2004; Bailey 2010). Supplementation with written educational materials was found to reduce the anxiety level of patients. In particular, when written content about the procedure and postoperative pain and nausea management were included (Kiyohara et al. 2004). This correlates with the first need of patients mentioned above and is also a point for service improvement (Bailey 2010; Hughes 2002; Spalding 2003). Our results show that information given to patients about postoperative symptom management was found to be partially met.

Second, Hughes suggested that nurse communication after admission with the patient helps relieve anxieties (Hughes 2002). A top need for patients in our results is the need for reassurance just before surgery and if the operation is delayed. This need is related to a high level of anxiety before elective surgery (Jawaid et al. 2007).

Third, in concordance with the second- and third-ranked needs of family members, a systematic review suggests that a person to person progress report in the intraoperative period reduces the anxiety level of accompanying family members, especially if the surgical time is taking longer than expected (Dexter and Epstein 2001). Intraoperative progress report by phone also decreases the anxiety level and improves the satisfaction level of accompanying family members (Blum and Burns 2013).

Additionally, a systematic review was done to explore the various evidence-based approaches to tackle patients’ anxiety during the perioperative period listed that relaxation techniques and music therapy were effective in reducing anxiety (Bailey 2010).

Our surprising finding in this research is that unlike patients, family members highly ranked the need to be informed of surgical procedures. This is different from the previous study of Davis et al. (Davis et al. 2014) where the patients and their family members both ranked this need as the highest need. The literature suggests that preoperative education to patients about surgical procedure improves their anxiety but does not explore that on the family members’ side (Kiyohara et al. 2004). Having this need ranked highly by family members and not patients in our survey raises a similar question discussed in the introduction to the unique cultural setting in Saudi Arabia. It also raises questions about relevant factors involved such as age, gender, health status, educational level or other cultural influences. There is an indication that the family member in Saudi culture plays an essential role in the emotional support and decision making for the patient; however, research in this is limited (Halligan 2006; Saleh Al Mutair et al. 2014). We also noticed in our results that the majority of patients are older and have a lower educational level in comparison to family members, and these could play a factor in our results. Nevertheless, we do not know the patients’ perspectives on such family member’s need, and we do not know its impact on family members’ anxiety level here or in other cultures, which could be explored in further research.

We realize several limitations in our study. First, we could not generalize our result to other contexts and hospitals as it explores the characteristics of KAMC population of patients in a non-pilgrimage season time in a limited period. Second, the validity of the questionnaire from Davis et al. (Davis et al. 2014) was limited to content validity. Third, we did not explore the types of surgery these patients went through.

## Conclusions

In conclusion, in our study, we explored the needs and the satisfaction level of patients and accompanying family members in a tertiary and quaternary centre in Saudi Arabia. The results could be used as an impetus to co-produce patients and family-centred interventions. We would also like to explore how staff would perceive and prioritize patients’ and family members’ needs and compare them to the current results. Family members are an essential component of compatible cultural delivery of healthcare in Saudi; therefore, sensitivity to patients’ and family members’ needs is a competency when embraced would lead to better experience and outcome. Studies that explore the cultural context in Saudi Arabia particularly, autonomy in health-related issues related to age, gender and educational level and how to approach such differences professionally are needed, as western values in healthcare would need to be tailored. Future action research to evaluate the impact of initiatives on satisfaction and anxiety level, determining anxiety levels in patients and family members are recommended.

## Data Availability

All data generated or analysed during this study are included in this published article and its supplementary information files.

## Abbreviations

PACU: Postanesthesia care unit
KAMC: King Abdullah Medical City
